# Combining Satellite Tracking and Remote Sensing to Identify Activity Pattern and Habitat Selection of Coastal Shorebirds: A Case Study of Pied Avocets in Bohai Bay, China

**DOI:** 10.1002/ece3.71143

**Published:** 2025-03-16

**Authors:** Dong Li, Xiyong Hou, Kai Liu, Yingxu Gao, Yang Wu

**Affiliations:** ^1^ Yantai Institute of Coastal Zone Research Chinese Academy of Sciences Yantai Shandong China; ^2^ CAS Key Laboratory of Coastal Environmental Processes and Ecological Remediation, Yantai Institute of Coastal Zone Research Chinese Academy of Sciences Yantai Shandong China; ^3^ Shandong Key Laboratory of Coastal Environmental Processes Yantai Shandong China; ^4^ University of Chinese Academy of Sciences Beijing China; ^5^ Key Laboratory for Biodiversity Science and Ecological Engineering, College of Life Sciences Beijing Normal University Beijing China

**Keywords:** bird movement, breeding period, home range, spatial analysis, wetland

## Abstract

In the context of intense interference from human activities and massive loss of natural wetlands in coastal zones, it is crucial to understand the behavioral ecology of shorebirds for formulating targeted conservation measures. Based on satellite tracking and remote sensing techniques, our research investigated the activity pattern and habitat selection characteristics of the Pied Avocet ( *Recurvirostra avosetta* ) in Bohai Bay, China. The results showed that Pied Avocets are relatively flexible in habitat selection in Bohai Bay. There are obvious individual differences in their residence time (119–210 days) and activity range (core home range from 15.34 to 95.12 km^2^). Pied Avocets may only move around a fixed breeding site throughout the breeding season, or they may transfer to another location for a second breeding. The mariculture, salt pan, and industrial‐mining land are the main components of the Pied Avocets' habitat, and the proportion of their area in the main and core home ranges is > 10%. The species prefers sparsely vegetated areas near coastlines and water bodies as habitats and has a certain tolerance for human disturbance. Our findings could provide specific management advice for alleviating human‐bird conflicts in the highly developed coastal zones. The study on the activity pattern and habitat selection of the Pied Avocet offered technical and data support for shorebirds habitat protection.

## Graphical abstract

Activity pattern and habitat selection characteristics of the Pied Avocet in Bohai Bay, China were investigated based on satellite tracking and remote sensing techniques. Pied Avocets are relatively flexible in habitat selection in Bohai Bay. We provide valuable insights into the breeding ecology of the Pied Avocet, offering technical and data support for waterbirds habitat protection.

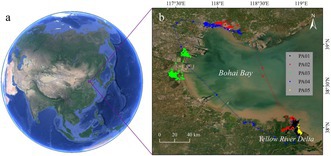

## 1 Introduction

Coastal wetlands play an important role in providing habitats for shorebirds in the world (Barbier et al. 2011); meanwhile, they are facing significant challenges from both natural disturbances and human stresses, like sea level rise, coastal erosion, land reclamation, invasive species, and reduced sediment flux from major rivers (Wang et al. 2021). Located in the center of the East Asia–Australasian Flyway (EAAF), China's coastal wetlands supply breeding, migratory stopover, and wintering grounds for tens of millions of waterbirds (Ma et al. 2023). However, they have experienced dramatic degradation, and half of the coastal wetlands have been lost in China due to intensive and large‐scale human activities in coastal areas from the 1950s to early 2000s (Ma et al. 2014). This has led to significant declines in many waterbird populations (Li et al. 2023; Lei et al. 2018; Zou et al. 2016). Therefore, the living conditions and habitat protection of coastal shorebirds have attracted extensive attention recently (Duan and Yu 2022; Lei et al. 2021; Wang et al. 2020).

It is of great significance to understand the activity pattern and habitat selection characteristics of shorebirds for their conservation. At present, satellite tracking and remote sensing technology have been applied more and more in the study of bird habitat use. For example, based on tracking and remote sensing, the critical year‐round site and habitat use of white‐naped cranes (*Antigone vipio*) were identified between their breeding grounds in eastern Mongolia and wintering areas in China's Yangtze River Basin (Batbayar et al. 2021). Also, the home range and habitat disturbance for Saunders's Gull (*Saundersilarus saundersi*) were distinguished in the Yellow River Delta, China (Xu et al. 2021). These methods are particularly useful in areas that are physically inaccessible. However, most related studies focus on flagship species, while those common species are often neglected (Barik et al. 2021), which hinders our knowledge of the potential risk the least concerned species may face during their life cycle (Wu et al. 2022). In fact, although common species may account for a small fraction of the species richness, they usually define the structure, character, and dynamics of ecosystems (Gaston 2010). Even a relatively small percentage decline in the abundances of common species can lead to negative impacts on critical ecosystem processes and services, including decomposition, pest control, and seed dispersal (Inger et al. 2015; Whelan et al. 2008). Therefore, considering the important role of common species in the ecosystem, it is particularly necessary to pay attention to their survival status.

The Pied Avocet ( *Recurvirostra avosetta* ) is a common shorebird, widely distributed in Eurasia and Africa. This species is a ground‐nesting, colonially breeding wader (Lengyel 2006). They breed in flat open areas with sparse vegetation and usually along shallow saline lakes, pools, saltpans, and estuaries (Hötker 2000), mainly feeding on small crustaceans, mollusks, ragworms, insect larvae, and small fish (Enners et al. 2019). They may reproduce twice in a single breeding season (Wu et al. 2022) and both sexes participate in incubation and chick rearing (Chokri and Selmi 2011). In the study of habitat suitability assessment of the Pied Avocet using the MaxEnt model in the Yellow River Delta (south of Bohai Bay), China, we made a preliminary understanding of the key environmental factors affecting the habitat selection for this species (Li, Xu, et al. 2024), but did not identify their activity pattern. In the study of habitat use of the Pied Avocet in an artificial wetland complex (Li, Li, et al. 2024), only an environmental variable (land use type) was analyzed, and no other environmental factors were involved. To conclude, in our previous studies, the activity pattern of the Pied Avocet in the whole Bohai Bay was little known, and their habitat preference was also poorly understood.

Based on satellite tracking and remote sensing techniques, this research takes the Pied Avocet in Bohai Bay as an example, aiming to achieve the following goals: (1) identifying their activity pattern during the breeding season; (2) understanding the habitat selection characteristics; and (3) proposing constructive suggestions for the protection of Pied Avocets as well as their habitats.

## 2 Materials and Methods

### 2.1 Study Area

**FIGURE 1:**
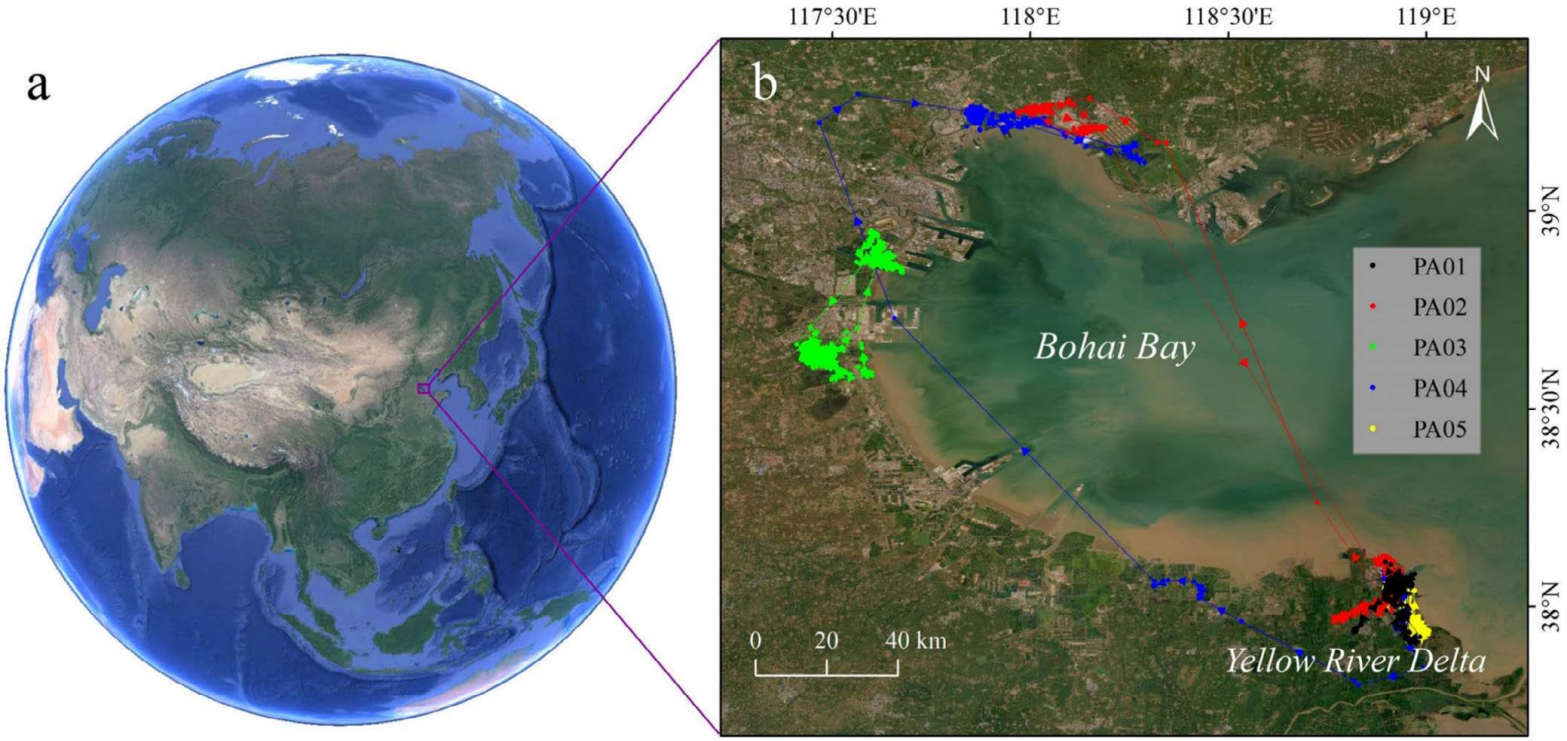
(a) The location of Bohai Bay, China; and (b) tracking records of the five tagged Pied Avocets in the study area.

Bohai Bay is a semi‐enclosed bay in northeastern China (Figure 1) and has a saline area larger than 2000 km^2^ (Shi et al. 2022). Located at the key node in the EAAF, the coast of Bohai Bay provides critical habitats for shorebirds (Yang et al. 2011). However, as one of the most important economic centers in North China, its coast is experiencing rapid industrialization (Zhu et al. 2016). Human disturbance and land use changes driven by reclamation have resulted in the loss and degradation of coastal wetlands in Bohai Bay, posing threats to waterbird populations (Wu et al. 2020). Many artificial wetlands, such as salt pans and mariculture ponds, have replaced a great deal of coastal natural habitats, becoming the alternative or sometimes indispensable foraging/breeding grounds for numerous shorebirds, including the Pied Avocet (Lei et al. 2021).

The Pied Avocet is a gregarious bird with an average incubation period of 24 days (Lei et al. 2021). They have been documented breeding in the saltpan complex (Lei et al. 2021) and abandoned oil exploitation area (Li, Li, et al. 2024) in Bohai Bay. On the southern coast of Bohai Bay, this species tends to forage in the pond and salt pan, with an unfixed staying time in the breeding ground (Li, Li, et al. 2024). It is reported that about 50% of nests failed during breeding on the northern coast of Bohai Bay, mainly because of rainfall and anthropogenic activities (Lei et al. 2021). Thus, the study of the Pied Avocet in Bohai Bay provides a fitting case for exploring the behavioral ecology of common shorebirds under intense interference from human activities.

### 2.2 Satellite Tracking and Environmental Data

#### 2.2.1 Satellite Tracking Data

In June 2022, we fixed satellite tracking devices on five adult Pied Avocets in the Yellow River Delta in Bohai Bay. The backpack satellite tracker (HQBG1206, Hunan Global Messenger Technology, China) weighs 6.7 g, < 3% of the bird's body weight. After wintering in southern China, all five birds migrated back to Bohai Bay to breed in 2023. This study focused on their movements from the time they arrived in Bohai Bay in 2023 to the time they left. The five shorebirds stayed in Bohai Bay for different periods of time (Table 1). Their activity information was recorded by satellite trackers every 2–6 h, including time, longitude, latitude, movement speed, and positioning accuracy. The position data accuracy index was categorized into five class levels, that is, A (5 m), B (10 m), C (20 m), D (100 m), and E (2000 m). In this research, the recorded data with positioning accuracy above class C, that is, the location error < 20 m, were retained, and finally, there were a total of 6667 tracking points for further analysis (Figure 1b and Table 1).

**TABLE 1:** Statistics of the five Pied Avocets' tracking points in the Bohai Bay.

Bird ID	Arrival time	Departure time	Duration of stay (days)	No. of tracking points
PA01	2023‐05‐26	2023‐09‐21	119	1054
PA02	2023‐03‐27	2023‐10‐20	208	1332
PA03	2023‐05‐02	2023‐11‐27	210	1452
PA04	2023‐04‐06	2023‐08‐24	141	1157
PA05	2023‐04‐01	2023‐10‐19	202	1672

Considering the living habits of Pied Avocets and data availability, combined with previous related literatures (Lei et al. 2018; Li, Xu, et al. 2024; Wu et al. 2022), we selected the following environmental variables to analyze the habitat preference of this species.

#### 2.2.2 Land Use and Land Cover (LULC) Data

The land use and land cover (LULC) data of the study area was updated in 2023 based on the China Coastal Land Use Database (2020), which was acquired by visual interpretation on the basis of Landsat images, with an overall cartographic accuracy of 94.12% and a Kappa coefficient of 0.92 (Du et al. 2022). The data have a spatial resolution of 30 m and distinguish 24 LULC types, including 12 types of wetlands.

#### 2.2.3 NDVI and NDWI Data

The normalized difference vegetation index (NDVI) is an important indicator of vegetation coverage, with the value ranging from −1 to 1, and it can reflect the shelter of waterbirds habitat to a certain extent (Li, Xu, et al. 2024; Teng et al. 2023). A positive value suggests that there is vegetation coverage, and the closer it is to 1, the greater the vegetation coverage. The normalized difference water index (NDWI) can reflect the hydrological conditions of waterbird habitat (Teng et al. 2021). It has been proven that waterbird species abundance is significantly correlated with NDWI in wetlands (Malekian et al. 2022). The value of NDWI is also from −1 to 1, and the larger the NDWI, the larger the water area. With the help of the Google Earth Engine (GEE) platform, we calculated the average NDVI and NDWI of the activity period within the home range of the tracked shorebirds using time series Sentinel‐2A remote sensing images with a spatial resolution of 10 m. They were calculated according to the following formulas:

(1) $$\text{NDVI}\;=\;\frac{\mathrm{NIR}\;-\;\mathrm{RED}}{\mathrm{NIR}\;+\;\mathrm{RED}}$$

(2) $$\text{NDWI}\;=\;\frac{\mathrm{GRE}\;-\;\mathrm{NIR}}{\mathrm{GRE}\;+\;\mathrm{NIR}}$$

where *NIR*, *RED*, and *GRE* are the reflectance of near‐infrared, red, and green wavelengths, according to band 8, band 4, and band 3 of Sentinel‐2A, respectively.

#### 2.2.4 Other Environmental Variables Data

The distance between the shorebirds' habitat and coastline, water body, and human disturbance can mirror their environmental preferences to a large extent. The coastline data in 2023 was updated according to the Chinese coastline (2020), obtained by visual interpretation using Landsat images (Xu et al. 2023). Water bodies and human disturbance information were extracted from the LULC data. The water body included paddy, rivers, lakes, reservoir/pond, estuarine waters, and estuarine delta, while human disturbance factors mainly considered man‐made features, such as urban area, rural settlement, road, and isolated industrial‐mining.

### 2.3 Home Range Identification

Home range is defined as “that area traversed by the individual in its normal activities of food gathering, mating and caring for young” (Burt 1943), and several calculation methods for home range have been developed in recent decades. In this research, the widely used kernel density estimation (KDE) method (Worton 1989) was applied to determine the home range of each tagged individual. The kernel density estimator for bivariate data is mathematically defined as (Seaman and Powell 1996)

(3) $$\overset{⌢}{f}\left(x\right)\;=\;\left[\frac{1}{{\mathrm{hh}}^{2}}\right]\;\sum_{i=1}^{n}K\;\left\{\frac{x-X_{i}}{h}\right\}$$

Where *n* is the number of tracking points, *h* indicates a kernel bandwidth, *K* means a kernel density, *x* is a vector of *x*, *y* coordinates describing the location where the function is being evaluated, and *X*_*i*_ suggests a series of vectors whose coordinates describe the location of each observation *i*.

The Home Range Tools (HRT) software in ESRI ArcGIS (Rodgers et al. 2015) was used to identify the Pied Avocets' home ranges based on a fixed kernel estimator, and the plug‐in estimator (Zhang et al. 2022) was applied to evaluate a kernel smoothing parameter for each individual (see Table S1). The density isopleth values of 95% and 50% were chosen as the boundaries of the main and core home ranges according to previous research (Dayananda et al. 2024; Dykstra et al. 2001; Martinez‐Miranzo et al. 2016).

### 2.4 Habitat Selection Analysis

The analysis of Pied Avocets' habitat selection was carried out based on the ArcGIS platform. The home ranges of shorebirds and LULC data were superimposed spatially, then the LULC types and their areas within the home ranges were counted and compared. LULC type, NDVI and NDWI value of the location of tracking points were obtained using the “Extract Multi Values to Points” tool embedded in the spatial analyst toolkit. Besides, distance to coastline, distance to water body and distance to human disturbance of tracking points were calculated by means of “Proximity” tool in the analysis toolkit.

## 3 Results

### 3.1 Activity Pattern of the Tagged Pied Avocets Along Bohai Bay

**FIGURE 2:**
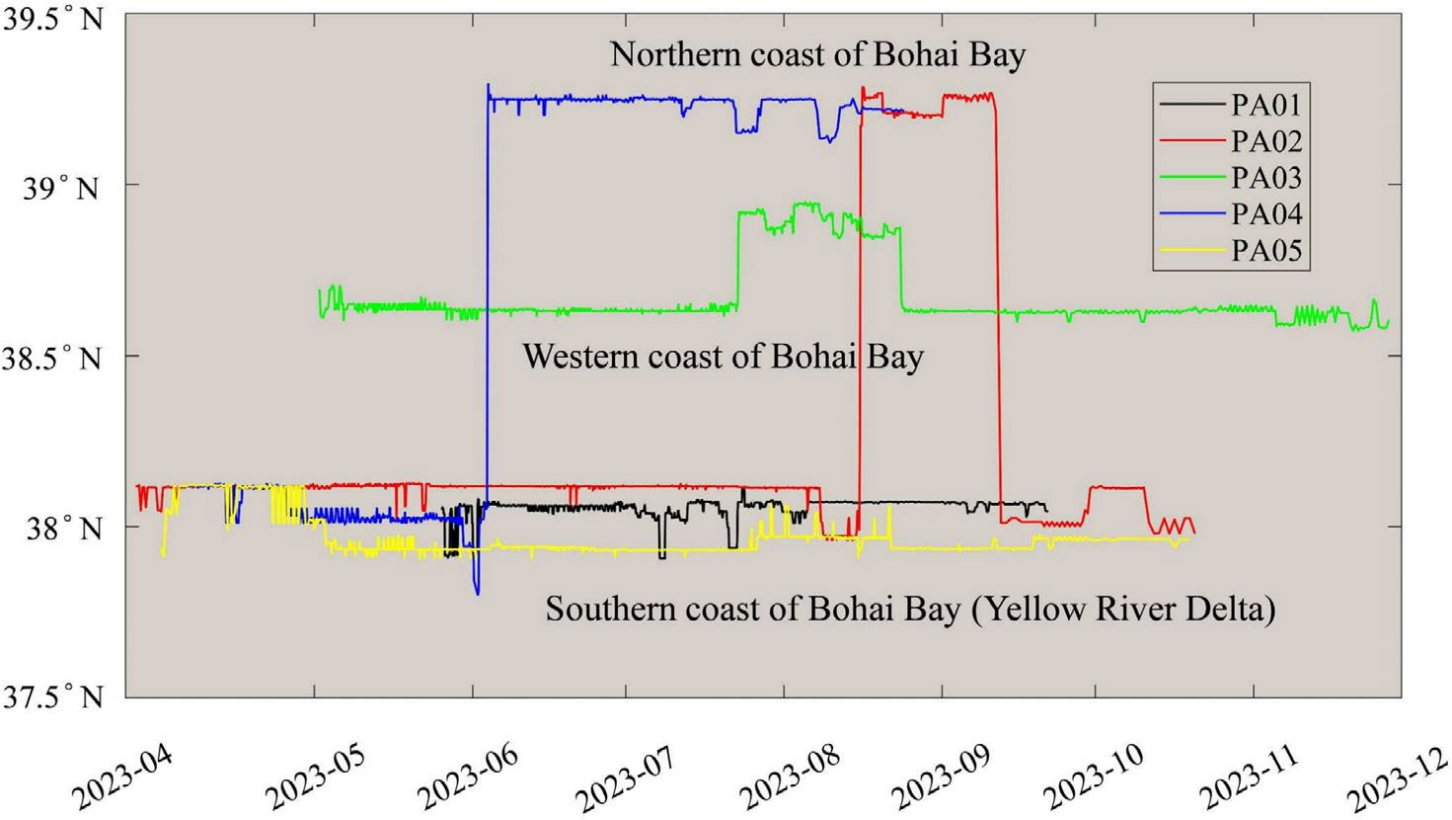
Latitudinal movements of the five Pied Avocets on the coast of Bohai Bay in 2023.

**FIGURE 3:**
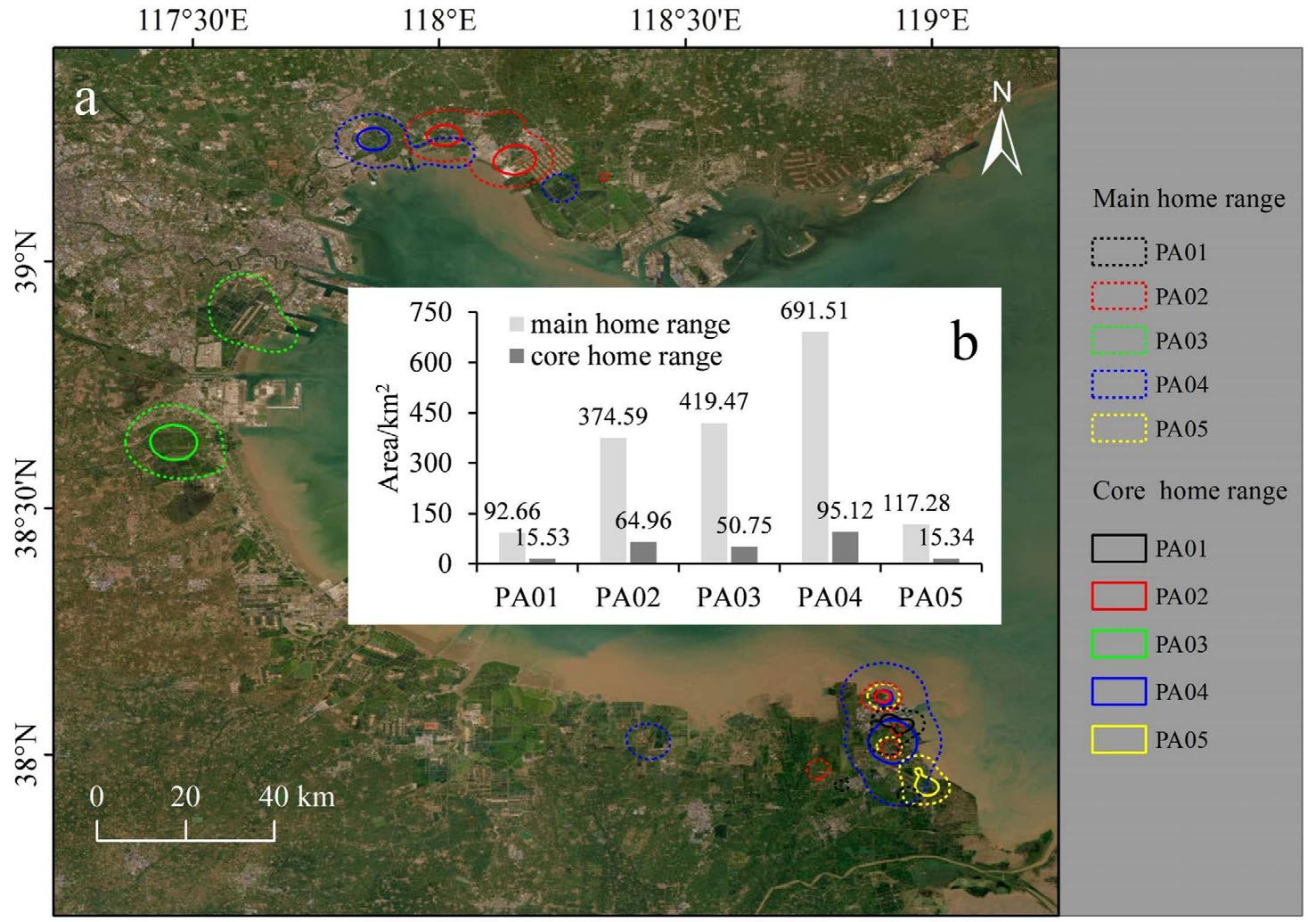
(a) Distribution of the five Pied Avocets' home ranges; and (b) comparison of individual main and core home ranges.

Pied Avocets showed individual differences in their activity patterns and home ranges in Bohai Bay (Figures 2 and 3). The individual PA01 arrived in the Yellow River Delta in the southern part of Bohai Bay on May 26, 2023, and stayed there for nearly 4 months until leaving on September 21, 2023. It had the smallest activity area among the five birds, and the main and core home range areas were 92.26 km^2^ and 15.53 km^2^, respectively. The Pied Avocet PA02 reached the Yellow River Delta on March 27, 2023, stayed for 142 days, and flew across Bohai Bay to its northern coastal area on August 15, 2023. It remained there for < 1 month (26 days), flew back across Bohai Bay to the Yellow River Delta on September 11, 2023, staying there for more than 1 month, and finally migrated south on October 20, 2023. The total main and core home range area of PA02 in the south and north sides of Bohai Bay was 374.59 km^2^ and 64.96 km^2^, respectively. The individual PA03 reached the western coastal zone of Bohai Bay on May 2, 2023, stayed for 82 days, and moved to the area about 30 km northeast of the original activity zone on July 23, 2023, staying there for 1 month. However, it returned to the original area on August 23, 2023, stayed for more than 3 months, and finally left Bohai Bay on November 27, 2023. Its total main and core home range in the two areas were 419.47 km^2^ and 50.75 km^2^, respectively. PA04 got to the Yellow River Delta on April 6, 2023, began moving along the coast of Bohai Bay on May 30, 2023, and reached the northern coastal zone 5 days later. After staying there for 81 days, it left Bohai Bay, migrating to the south. It had the largest activity area among the five birds, and the total main and core home range area in the south and north sides of Bohai Bay were 691.51 km^2^ and 95.12 km^2^, respectively. Similar to PA01, PA05 did not move long distances after reaching the Yellow River Delta. It stayed there between April 1, 2023 and October 19, 2023. The main and core home range of PA05 were 117.28 km^2^ and 15.34 km^2^, respectively.

### 3.2 Habitat Selection of Pied Avocets

#### 3.2.1 LULC Within the Home Range

**FIGURE 4:**
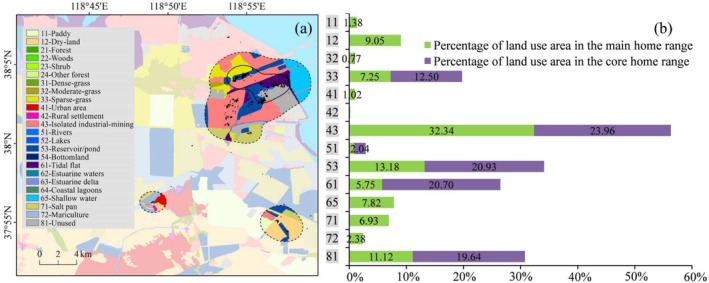
Different land use and land cover (LULC) types (a) and their area proportions (b) in the main and core home range of individual PA01. The legend of LULC types is on the left side of the figure (a), which is described in Table S2.

**FIGURE 5:**
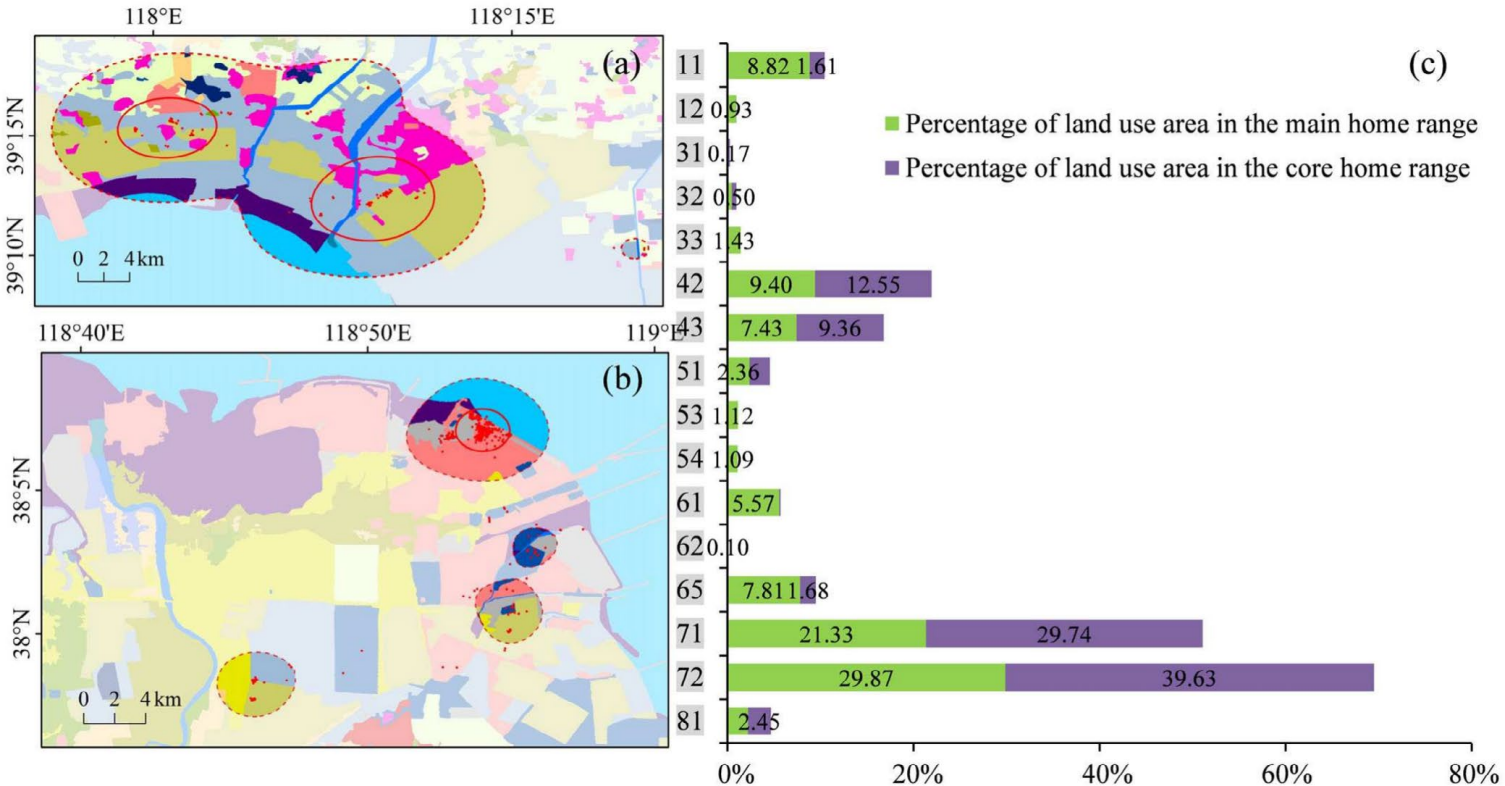
Different LULC types (a and b) and their area proportions (c) in the main and core home range of individual PA02. The LULC type legend is consistent with Figure 4a.

**FIGURE 6:**
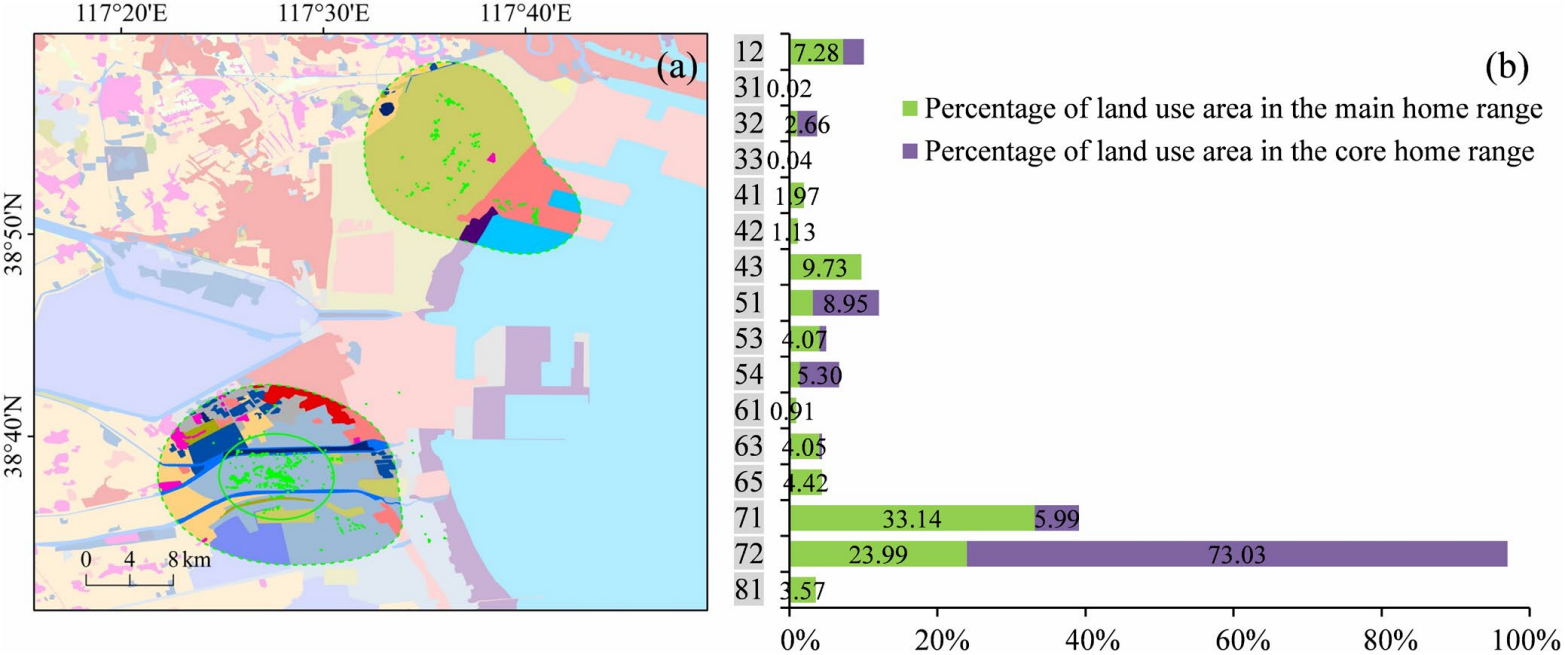
Different LULC types (a) and their area proportions (b) in the main and core home range of individual PA03. The LULC type legend is consistent with Figure 4a.

**FIGURE 7:**
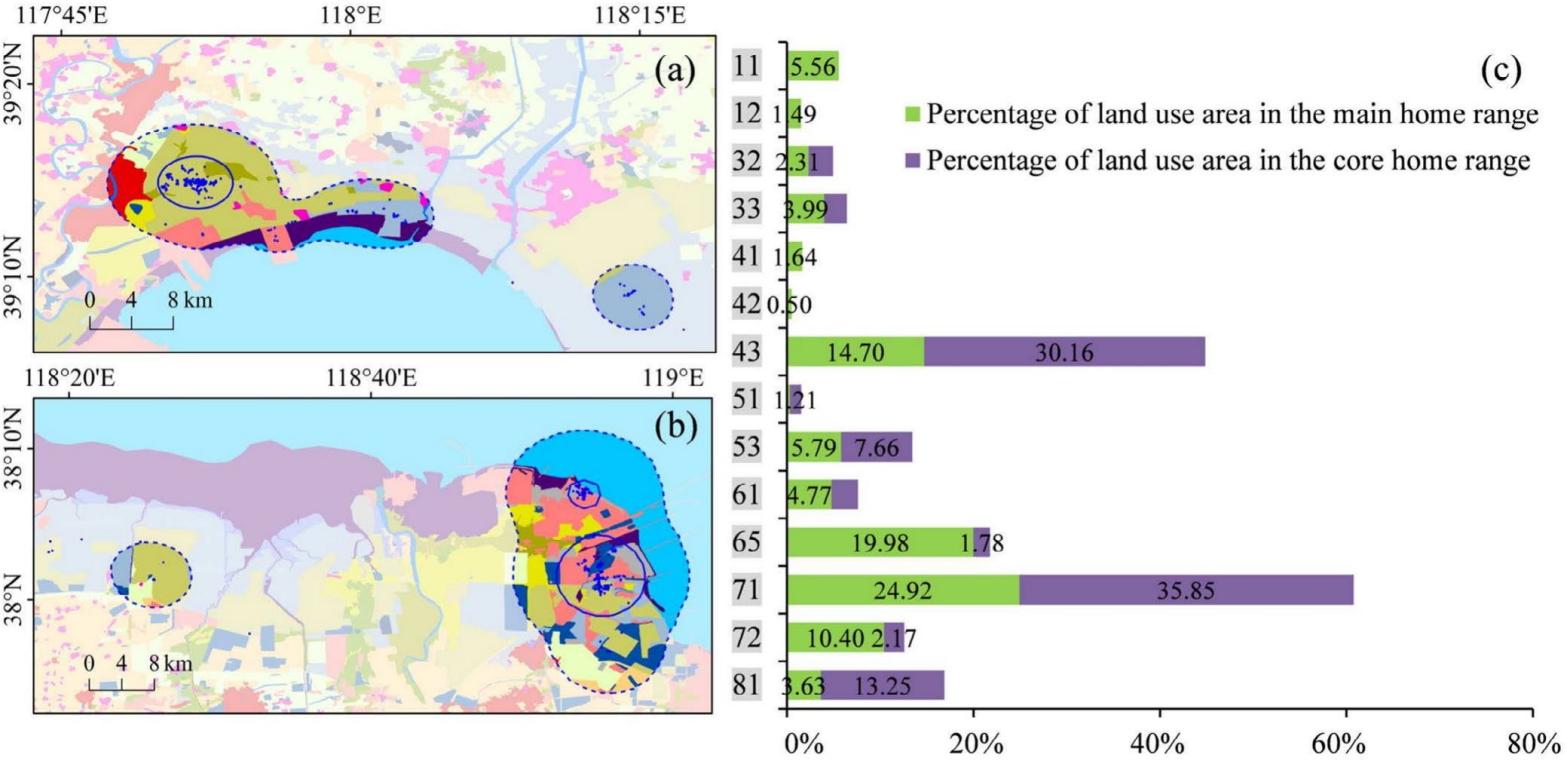
Different LULC types (a and b) and their area proportions (c) in the main and core home range of individual PA04. The LULC type legend is consistent with Figure 4a.

**FIGURE 8:**
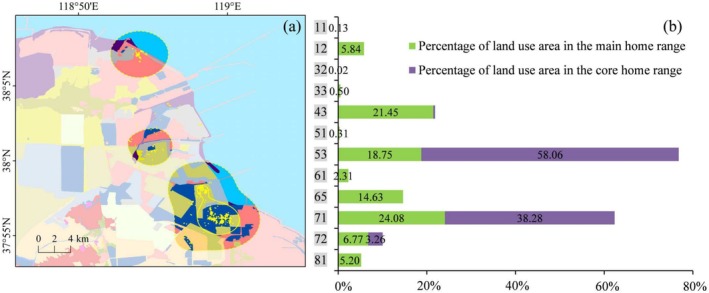
Different LULC types (a) and their area proportions (b) in the main and core home range of individual PA05. The LULC type legend is consistent with Figure 4a.

The LULC type and area proportion in the main and core home range of the five tagged Pied Avocets are displayed in Figures 4, 5, 6, 7, 8.

The top three LULC types in the main home range of PA01 were isolated industrial‐mining, reservoir/pond, and unused land, whose total area proportion accounted for 56.64%. In PA01's core home range, the top three types were industrial‐mining, reservoir/pond, and tidal flat, accounting for 65.59%. The LULC types that accounted for more than 20% in the main home range of PA02 were mariculture (29.87%) and salt pan (21.33%). The situation of PA02's core home range was similar to that of the main home range, with mariculture and salt pan accounting for 39.63% and 29.74%, respectively. The two types of LULC, mariculture and salt pan, were also dominant in PA03's activity area, with the proportion of 57.13% in the main home range and as high as 79.02% in the core home range. The LULC types that represented more than 10% in the main home range of PA04 included salt pan (24.92%), shallow water (19.98%), isolated industrial‐mining (14.70%), and mariculture (10.40%); while in the core home range, the LULC types contributing over 10% were salt pan (35.85%), isolated industrial‐mining (30.16%), and unused land (13.25%). There were four primary LULC types accounting for nearly 80% of the main home range of PA05, that is, salt pan (24.08%), isolated industrial‐mining (21.45%), reservoir/pond (18.75%), and shallow water (14.63%). In PA05's core home range, the area of the two LULC types, reservoir/pond and salt pan, occupied more than 96%.

Further, the use of natural and artificial wetlands by the tracked Pied Avocets was analyzed (Table 2). Here, natural wetlands include rivers, bottomland, tidal flat, estuarine waters, estuarine delta, and shallow water; artificial wetlands include paddy, reservoir/pond, salt pan, and mariculture. Among the five individuals, PA02 exhibited the highest proportion of wetland area within the main home range at 79.71%, and PA05 showed the highest within the core home range at 99.59%. According to the statistics, the use of artificial wetland has substantially exceeded that of natural wetland in their home ranges. Within the main home range, the maximum ratio of artificial wetlands proportion to natural wetlands appeared in PA03, reaching 4.06. It was followed by PA02, PA05, PA04, and PA01, which were 3.29, 2.87, 1.76, and 1.63, respectively. Within the core home range, the proportion of artificial wetlands was significantly greater than that of natural wetlands in all the five individuals except PA01. The core home range of PA05 was completely composed of artificial wetlands, and the ratios of artificial wetlands proportion to natural wetlands were 17.53, 7.60, and 5.31 for PA02, PA04, and PA03, respectively.

**TABLE 2:** Comparison of the natural and artificial wetlands in the main home range (M_HR) and core home range (C_HR) of the five Pied Avocets.

	PA01		PA02		PA03		PA04		PA05	
	M_HR	C_HR	M_HR	C_HR	M_HR	C_HR	M_HR	C_HR	M_HR	C_HR
Natural wetlands	13.35 km^2^ (14.61%)	3.25 km^2^ (20.93%)	63.42 km^2^ (18.57%)	2.59 km^2^ (4.05%)	58.58 km^2^ (15.06%)	7.42 km^2^ (15.04%)	173.30 km^2^ (26.54%)	5.57 km^2^ (6.01%)	20.23 km^2^ (17.27%)	0.00 km^2^ (0.00%)
Artificial wetlands	22.12 km^2^ (23.87%)	3.53 km^2^ (22.79%)	229.04 km^2^ (61.14%)	46.11 km^2^ (70.99%)	256.71 km^2^ (61.20%)	40.56 km^2^ (79.93%)	322.75 km^2^ (46.67%)	43.44 km^2^ (45.67%)	58.32 km^2^ (49.73%)	15.28 km^2^ (99.59%)

**FIGURE 9:**
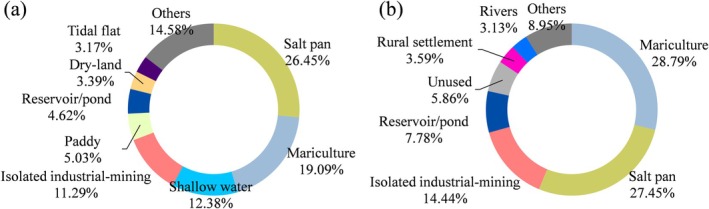
Area proportion of different LULC in the combined main home range (a) and core home range (b) for five Pied Avocets in Bohai Bay, China.

**FIGURE 10:**
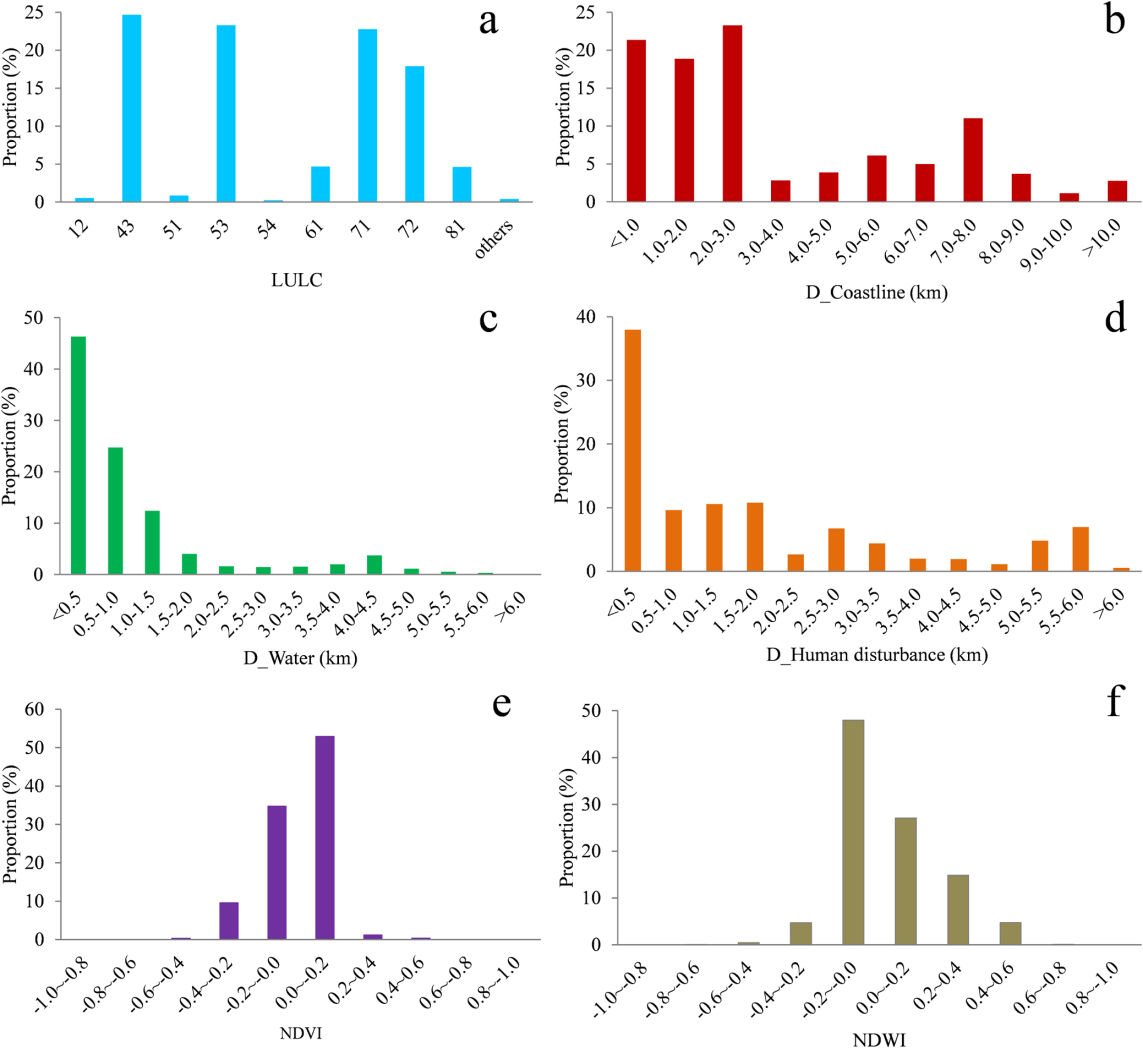
Environmental variable characteristics of tracking points. (a) Land use and land cover (LULC), (b) distance to coastline, (c) distance to water body, (d) distance to human disturbance, (e) normalized difference vegetation index (NDVI), and (f) normalized difference water index (NDWI). The labels on the *x*‐axis of figure (a) indicate LULC types, and the number corresponds to its meaning in Figure 4a and Table S2.

**FIGURE 11:**
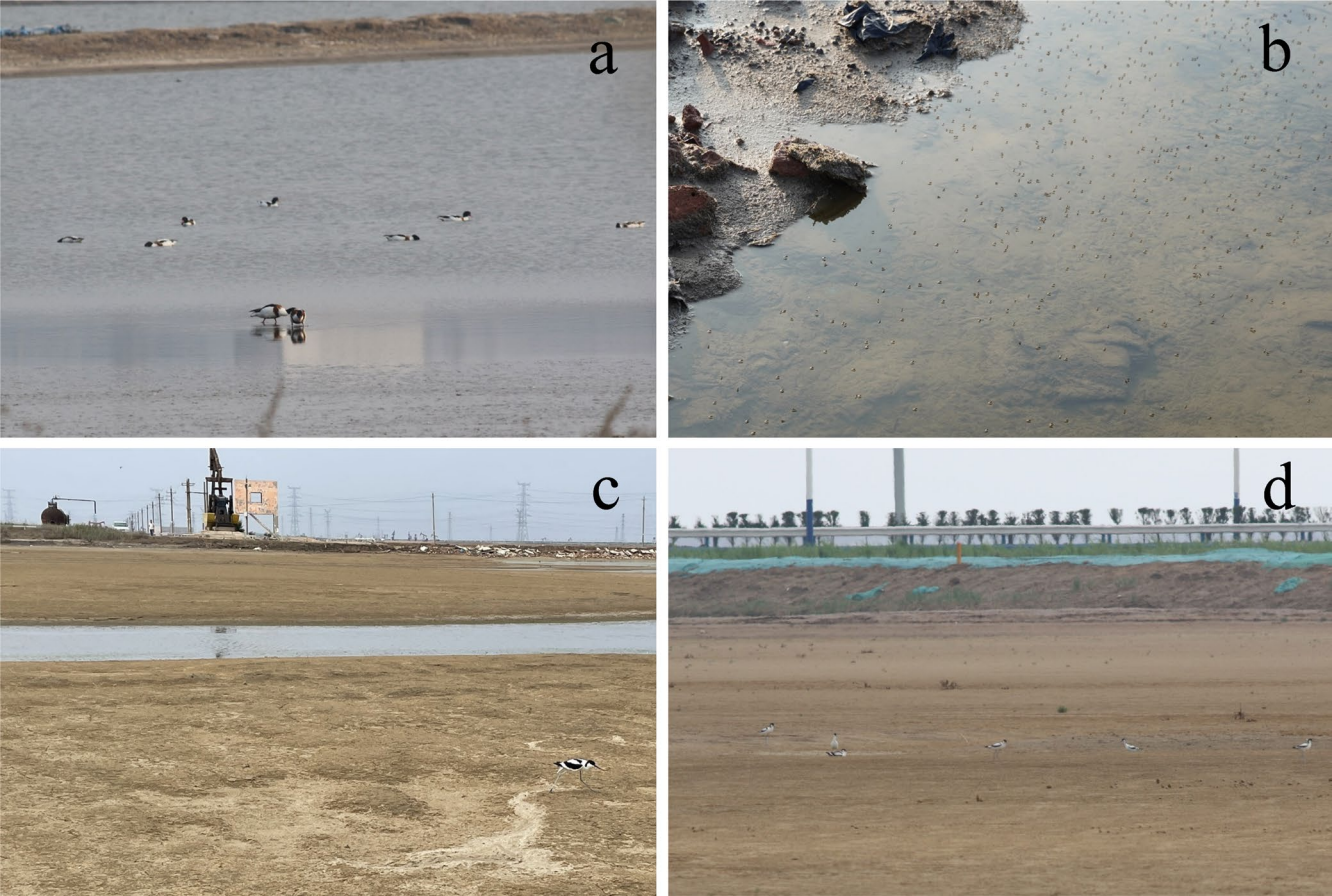
Photos of Pied Avocets feeding in a shrimp pond (a), brine flies in the salt pan (b), the semi‐natural wetland used as breeding grounds by the Pied Avocet (c), and Pied Avocets' close to the road (d), respectively.

When all tagged Pied Avocets' home ranges were spatially combined, the results showed that the total main home range and core home range were 1421.38 km^2^ and 227.06 km^2^, respectively. The area proportion of different LULC types in the combined main and core home range is shown in Figure 9. It can be seen that the three LULC types, mariculture, salt pan, and isolated industrial‐mining land were dominant both in the main home range and core home range, all of their area proportions > 10%. In the main home range, salt pan had the largest area proportion, reaching 26.45%. It was followed by mariculture (19.09%), shallow water (12.38%) and isolated industrial‐mining land (11.29%). These four types of LULC accounted for nearly 70% of the main home range. In the core home range, mariculture (28.79%) and salt pan (27.45%) accounted for more than half of the area, followed by isolated industrial‐mining land (14.44%). In addition, the reservoir/pond occupied a certain proportion in both home ranges: 4.62% in the main home range and 7.78% in the core home range.

#### 3.2.2 Environmental Characteristics of the Tracking Points

The statistical results of environmental variables for tracking points of the five tagged Pied Avocets are shown in Figure 10. From the perspective of LULC type, the Pied Avocet favored isolated industrial‐mining, reservoir/pond, salt pan, and mariculture, and the proportions of tracking points in these four lands were 24.68%, 23.31%, 22.76%, and 17.91%, respectively (Figure 10a). It can be seen that Pied Avocets took semi‐natural wetlands and artificial wetlands as their main habitat. In terms of distance to coastline, 63.53% of the tracking points were located within 3 km of the coastline, indicating that the Pied Avocet favored areas close to the coastline (Figure 10b). From the angle of distance to water body, 46.31% of the tracking points were distributed within 500 m of a water body, and more than 90% within 3 km (Figure 10c), suggesting this species preferred habitats near a water body. From the point of distance to human disturbance, 37.98% of the tracking points were within 500 m of the human disturbance, and 78.32% within 3 km (Figure 10d), meaning the species could tolerate a certain amount of human interference. There were 87.98% of the tracking points with NDVI values from −0.2 to 0.2, indicating that Pied Avocets always inhabited areas with sparse vegetation. Meanwhile, more than 75.09% of the tracking points had NDWI values from −0.2 to 0.2, showing they tended to select wet habitat to forage, rest, or breed.

## 4 Discussion

### 4.1 Activity Pattern of Pied Avocets Along Bohai Bay

Pied Avocets showed different residence periods in Bohai Bay. In our research, the earliest and latest arrival times of these five individuals in Bohai Bay in 2023 were in late March (PA02) and late May (PA01), whereas the earliest and latest departure times were in late August (PA04) and late November (PA03). They spent an average of 176 days in Bohai Bay, with the longest being 210 days (PA03). Given that the Pied Avocet is a gregarious shorebird species (Chambon et al. 2018) and there is no significant difference between the sexes in migration timing (Wu et al. 2022), it can be concluded that the species has a relatively flexible migration schedule. Unlike long‐distance migratory birds, such as the white‐naped crane (*Antigone vipio*) (Batbayar et al. 2021), Swan Goose ( *Anser cygnoides* ) (Damba et al. 2021), and Chinese Egret ( *Egretta eulophotes* ) (Huang et al. 2022), Pied Avocets' migration times are not as strict, most likely due to their low requirements for breeding and wintering grounds. This is also an important reason why this species is widely distributed in Eurasia and Africa, and is listed as Least Concern (LC) by the IUCN.

The coastal zone of Bohai Bay provides certain suitable habitats for Pied Avocets. In this research, using satellite tracking technology, different activity zones of the Pied Avocet were recognized in the southern, western, and northern coasts of Bohai Bay (Figure 3a). Once the Pied Avocet finds a suitable place to start breeding, its activity area is limited (Li, Li, et al. 2024), which was further confirmed in this study. For example, PA01 and PA05 spent their whole breeding period in the Yellow River Delta in the south of Bohai Bay, and their core home ranges were 15.53 km^2^ and 15.34 km^2^, respectively (Figure 3b). Similarly, PA03 also spent its whole breeding period in the same area, but on the west coast of Bohai Bay with a core home range of 50.75 km^2^ (Figure 3b). The small home range of Pied Avocets during the breeding period is closely related to their physiological needs. Their breeding system is seasonally monogamous, with both sexes performing egg incubation and chick brooding (Chokri and Selmi 2011). During the incubation and nursing stages, parental birds need to travel frequently between breeding and feeding grounds (Li, Li, et al. 2024), objectively limiting their activities to a range not far from the breeding grounds. This has also been observed in the Wadden Sea, where the Pied Avocet performed short foraging trips (maximum distance: 0.3–5.9 km) during the breeding season (Enners et al. 2019).

It is worth noting that both PA02 and PA04 resided on the southern and northern coasts of Bohai Bay for a period of time (Figures 2 and 3a). This has a lot to do with the breeding habits of the species. Many avocets can lay a second brood (Wu et al. 2022), so one possible explanation for this movement within the bay area is that the Pied Avocet tried to rebreed in northern Bohai Bay after their reproduction in the south. Differing from species with high breeding site fidelity, the Pied Avocet generally adopts an opportunistic ‘trial‐and‐error’ strategy (Lengyel 2006) by promptly building colonies where temporarily suitable for nesting and feeding. However, many factors such as flooding of nests, competition for nest sites by other species, nest predation, adverse weather, and abundance of food for chicks (Hötker and Sebebade 2000) will affect breeding success during both the laying and incubation periods. Thus, another reason they did not stay in one place during the breeding season may be related to reproduction failure at the original breeding site. High nest‐abandonment rates have been recorded for Pied Avocets in Bohai Bay mainly due to flooding and a lack of high‐quality breeding sites (Lei et al. 2021). However, PA02 did not complete the second reproduction on the north coast of Bohai Bay because it stayed there for < 1 month before flying back to the southern coast.

### 4.2 Selection of Environmental Factors by Pied Avocets

It is well known that food, water, shelter, and human disturbance are important factors in wildlife habitat selection. Previous studies have confirmed that LULC type is the key environmental variable to affect the habitat selection of birds (Chang et al. 2022; Na et al. 2018; Yamada et al. 2019). To a certain extent, LULC type determines whether it is suitable for nesting and whether it can provide enough food. In our research, we found that three LULC types, mariculture, salt pan, and isolated industrial‐mining land, accounted for the largest proportion of Pied Avocets' home ranges (Figure 9). The importance of coastal mariculture for waterbirds as feeding and roosting habitats is increasingly being recognized in the world, such as in the Inner Gulf of Thailand (Green et al. 2015), Yangtze River Estuary (Zou et al. 2016), north‐west Mexico (Fonseca and Navedo 2020), and the south coast of the United Kingdom (Clarke et al. 2019). Mariculture in Bohai Bay primarily raises shrimp with good economic benefits, providing a rich food source for avocets (Figure 11a). Meanwhile, it also provides alternative resting grounds for large numbers of shorebirds during high tide (Li et al. 2023). Salt pans can not only supply feeding grounds but also breeding sites. It is reported that the salt pan complex in northern Bohai Bay accommodated considerable numbers of Pied Avocets (Lei et al. 2021). With diverse dikes and islets, salt pans can provide suitable nest sites, and there are abundant food resources, such as chironomid larvae, *Artemia* spp., and brine fly larvae for the Pied Avocet to feed (Figure 11b). Notable is the high use of isolated industrial‐mining land by Pied Avocets in our study. This area was mainly a semi‐natural wetland slowly formed after the completion of human industrial activities such as oil extraction, mostly found in the Yellow River Delta in the southern part of Bohai Bay. It is most evident in the home range of individual PA01 (Figure 4), where the isolated industrial‐mining land occupied the largest proportion of all LULC types. This particular wetland, with less human disturbance, was chosen by Pied Avocets because it usually has tidal access to the sea, providing a fine place to breed and forage (Figure 11c). The total proportion of satellite tracking points in mariculture, salt pan, and isolated industrial‐mining land has reached 65.35% (Figure 10a), further proving the preference of the Pied Avocet for these three LULC types.

As a kind of shorebird living in the coastal zone, the Pied Avocet has a certain dependence on the saline‐alkali environment shaped by seawater for breeding and foraging. This was confirmed by the high proportion (more than 60%) of the tracking points located within 3 km of the coastline (Figure 10b). Remote sensing results showed that the Pied Avocet preferred areas with low NDVI values (Figure 10e), and our field survey in June 2022 further found that they mostly bred in saline‐alkali land with sparse vegetation (Figure 11c). Such a type of environment could give them a wide field of view to spot predators, increasing reproductive success (Li, Xu, et al. 2024). The Pied Avocet is a wading bird, and it often uses the typical upward‐curved bill to forage in muddy sediments (Moreira 1995). Therefore, they usually live in wetland areas such as swamps or watersides. This was validated in our study, where 75.09% of the tracking points had the NDWI values between −0.2 and 0.2 (Figure 10f). Areas of this index range typically have small water cover or shallow water levels, making it easier for Pied Avocets to access bottom‐dwelling invertebrates or other food resources.

In addition, because Bohai Bay is one of the areas where human exploitation activities are most intensive in China's coastal zone, a large number of shorebird habitats have been disturbed by human activities, like tidal flat reclamation, resource exploitation, and road construction (Figure 11d). In our study, more than three‐quarters of the tracking points are within 3 km of human disturbance (Figure 10d). It can be speculated that in the context of human activities encroaching on the natural habitat of shorebirds, many of them have to endure human interference to survive.

### 4.3 Implications for Sshorebird Habitat Conservation

Effective conservation requires diversified strategies tailored to the specific requirements of different waterbird species (Gao et al. 2025). Based on the information gained from our research, the following constructive suggestions are put forward for the habitat conservation of coastal shorebirds. First, it is suggested to reduce human disturbance to shorebird breeding grounds. Shorebirds face many challenges in the process of hatching and nursing (Chyb and Minias 2022), such as physical fatigue, extreme climate, and food shortage; therefore, human activities with a negative effect should be avoided around their breeding ground, providing them with a safe and low‐disturbance breeding environment. Second, greater emphasis should be placed on the habitat function of artificial wetland complexes. It is possible to establish an operational mechanism that balances production activities and habitat maintenance in artificial wetlands, such as adjusting the water level of some salt pans or aquaculture ponds (Cheng et al. 2022; Green et al. 2015) during the breeding season to provide more feeding grounds to meet shorebirds' energy needs. Third, considering the trend of massive habitat loss, it is high time that shorebird habitat protection must be incorporated into the coastal zone development planning. Government departments, scientific research institutions, and non‐governmental organizations need to harness their respective advantages to form a joint force to effectively protect coastal shorebird habitat in terms of data and technology support, scientific planning, policy publicity, and science popularization.

### 4.4 Research Limitations and Future Work

Limited by the tracker's power supply and the accuracy of the data positioning, the time interval of valid tracking points obtained in our study is a little long, one point every 3.17 h on average. The connection of two consecutive data points cannot accurately depict the birds' actual movement trajectories within such a long time interval. So our tracking data is not suitable for particularly detailed work, such as studying the activity features of the bird during the tide cycle and the movement trajectories between the nesting and feeding areas. However, considering the gregariousness of the waterbirds, limited tagged individuals and tracking points can reflect their behavioral ecology features to some extent (Ji‐Yeon et al. 2023; McKellar and Clements 2023; Wang et al. 2022). Although the tracking sample is limited, our study obtained Pied Avocets' activity patterns and habitat selection characteristics during the breeding season, providing a basis for formulating targeted conservation measures.

It is a prerequisite for the protection of shorebirds to master their activity patterns and habitat utilization characteristics. In the future, we will aim at increasing the sample number of tracked individuals to keep gaining insights into the movements and habitat selection of shorebirds. Besides, we will also build niche models based on species occurrence points and environmental variables to simulate and predict the suitable habitat range of shorebirds. It is expected to provide data and technical support for alleviating human‐bird conflicts in Bohai Bay and even the whole coastal zone of China.

## 5 Conclusions

In this research, the activity pattern and habitat selection characteristics of coastal Pied Avocets were identified in Bohai Bay, China, using satellite tracking and remote sensing techniques. Individual differences in the timing of their arrival and departure from Bohai Bay suggest that this species has a flexible migration strategy. During the breeding season, the Pied Avocet has a relatively small home range. However, they may change breeding grounds for a second brood in order to increase reproductive success. In Bohai Bay, Pied Avocets mainly choose mariculture, salt pan, and isolated industrial‐mining land as their habitats. They prefer to live in wetland areas with sparse vegetation. In addition, the species has a certain tolerance for human interference. This study provides valuable insights into the behavioral ecology of shorebirds and has practical significance for promoting coastal shorebird protection.

## Author Contributions

**Dong Li:** conceptualization (lead), data curation (equal), investigation (supporting), methodology (lead), writing – original draft (lead). **Xiyong Hou:** data curation (equal), formal analysis (lead), funding acquisition (lead), supervision (lead), writing – review and editing (lead). **Kai Liu:** investigation (supporting), software (supporting), validation (equal), visualization (equal). **Yingxu Gao:** investigation (equal), validation (supporting). **Yang Wu:** investigation (equal), resources (supporting), validation (equal).

## Conflicts of Interest

The authors declare no conflicts of interest.

## Supporting information


Data S1:

Data S2:

Data S3:

Data S4:

Data S5:

Data S6:

Data S7:

Data S8:

Data S9:

Data S10:

Data S11:

Data S12:

Data S13:

Data S14:

Data S15:

## Data Availability

The tracking data are stored in Movebank (https://www.movebank.org/) under ID 5637513826, study “2023 YIC‐CAS Pied Avocets satellite tracking data.” The land use and land cover (LULC) data are available from the Big Earth Data Science Data Center, Chinese Academy of Sciences (https://data.casearth.cn/). The normalized difference vegetation index (NDVI) and normalized difference water index (NDWI) data used in this study are published as Supporting Information S1–, S14. The kernel smoothing parameters used for each bird and the definition of land use classification system are showed in Supporting Information S15.
