# Supplementary material for: Combining Satellite Tracking and Remote Sensing to Identify Activity Pattern and Habitat Selection of Coastal Shorebirds: A Case Study of Pied Avocets in Bohai Bay, China
Source: Ecol Evol. 2025 Mar 16;15(3):e71143. doi: 10.1002/ece3.71143 (PMC11911026; doi:10.1002/ece3.71143)
Supplement: Supplementary file 15 — Data S15: [file ECE3-15-e71143-s014.docx]

TABLE S1. The kernel smoothing parameters used for each individual

| Bird ID | Geographical location | Kernel parameters | Bandwidth (m)  LSCV (least squares cross validation) |
| --- | --- | --- | --- |
| PA01 | South coast of Bohai Bay | Adaptive Kernel | 1265 |
| PA02 | South coast of Bohai Bay | Adaptive Kernel | 1288 |
|  | North coast of Bohai Bay | Adaptive Kernel | 2888 |
| PA03 | West coast of Bohai Bay | Adaptive Kernel | 3390 |
| PA04 | South coast of Bohai Bay | Adaptive Kernel | 3669 |
|  | North coast of Bohai Bay | Adaptive Kernel | 2411 |
| PA05 | South coast of Bohai Bay | Adaptive Kernel | 1594 |

TABLE S2. Land Use Classification System and its Definition for China’s Coastal Zone

| **Level 1** | | **Level 2** | | **Description** |
| --- | --- | --- | --- | --- |
| **Code** | **Name** | **Code** | **Name** |  |
| 1 | Farmland |  |  | Lands for agriculture |
|  |  | 11 | Paddy | Farmlands with water resource guarantee and irrigating facilities using for rice growing |
|  |  | 12 | Dry-land | Lands for cultivating without irrigating facilities; dry croplands and lands growing vegetables |
| 2 | Forest |  |  | Lands growing trees including arbor, shrub, bamboo and lands for forestry use |
|  |  | 21 | Forest | Natural or man-made forest with canopy cover greater than 30% |
|  |  | 22 | Woods | Lands covered by trees with canopy cover between 10-30% |
|  |  | 23 | Shrub | Lands covered by trees less than 2 meters high, the canopy cover > 40% |
|  |  | 24 | Other forest | Lands such as tea-garden, orchid and non-grownup forest |
| 3 | Grassland |  |  | Lands covered by herbaceous plant with coverage grater than 5%, including shrub-grass for pasture and the woods with cover canopies less than 10% |
|  |  | 31 | Dense-grass | Grassland with canopy cover grater than 50% |
|  |  | 32 | Moderate-grass | Grassland with canopy cover between 20-50% |
|  |  | 33 | Sparse-grass | Grassland with canopy cover between 5-20% |
| 4 | Built-up |  |  | Lands used for urban and rural settlements and factories and transportation facilities |
|  |  | 41 | Urban area | Lands used for cities and counties |
|  |  | 42 | Rural settlement | Lands used for settlements in villages |
|  |  | 43 | Isolated industrial-mining | Lands used for factories, quarries, mining, oil-field slattern outside cities and lands for special uses such as transportation and airport |
| 5 | Inland freshwaters  (Freshwater wetlands) |  |  | Lands covered by natural fresh-water bodies or lands with facilities for irrigation and water reservation in inland area |
|  |  | 51 | Rivers | Lands covered by rivers including canals |
|  |  | 52 | Lakes | Lands covered by lakes |
|  |  | 53 | Reservoir/pond | Man-made facilities for water reservation |
|  |  | 54 | Bottomland | Lands between normal water level and flood level |
| 6 | Coastal saltwater  (Saltwater wetlands) |  |  | Wetlands on the coast with saline waters |
|  |  | 61 | Tidal flat | Lands between high tide level and low tide level |
|  |  | 62 | Estuarine waters | Permanent water of estuaries and rivers that being affected by tidal waters |
|  |  | 63 | Estuarine delta | Alluvial low plain in estuary area, usually composed of sandy island, sandbank and spit |
|  |  | 64 | Coastal lagoons | Brackish to saline lagoons with at least one relatively narrow channel connected to the sea |
|  |  | 65 | Shallow water (along coast) | Areas at low tide depth of 6 m below sea level, including gulfs and straits |
| 7 | Human made (saltwater) wetland | 71 | Salt pan | Salt exploitation sites on the shoals, usually including evaporation ponds, crystallizing ponds and ancillary facilities (an area of low land where sea water has evaporated to leave salt) |
|  |  | 72 | Mariculture | Ponds usually constructed and managed for commercial aquaculture production |
| 8 | Unused | 81 | Unused | Lands that is not put into practical use or difficult to use and has very sparse vegetation, such as [saline](http://dict.youdao.com/w/saline/) [land](http://dict.youdao.com/w/land/), bare soil, bare rock, and so on |
